# Selective Estrogen Receptor Modulators in COVID-19: A Possible Therapeutic Option?

**DOI:** 10.3389/fphar.2020.01085

**Published:** 2020-07-15

**Authors:** Alba Calderone, Francesco Menichetti, Ferruccio Santini, Luciano Colangelo, Ersilia Lucenteforte, Vincenzo Calderone

**Affiliations:** ^1^ Obesity and Lipodystrophy Center, Endocrinology Unit, University Hospital of Pisa, Pisa, Italy; ^2^ Department of Clinical and Experimental Medicine, University of Pisa, Pisa, Italy; ^3^ Department of Clinical, Internal, Anaesthesiologic and Cardiovascular Sciences, Sapienza University of Rome, Rome, Italy; ^4^ Department of Pharmacy, University of Pisa, Pisa, Italy

**Keywords:** SARS-CoV-2, COVID-19, antiviral, cytokine storm, estrogen receptor, selective estrogen receptor modulators

## Introduction

Male and female genders exhibit significant differences in the outcome of infective diseases caused by several viral pathogens. Along with behavioral or social factors which can affect the exposure to infection and the availability of therapies, it is widely accepted that genetic and physiological factors can markedly influence sex-related differences in immune responses. In particular, receptors for gonadal hormones are expressed in many immune cell types and, consistently, sex-related differences in immune function are likely to be strongly influenced by circulating sex steroid hormones (Klein and Huber, 2010).

Concerning coronaviruses, epidemiological data from SARS epidemic (severe acute respiratory syndrome caused by SARS-CoV in 2002–2003) and MERS epidemic (Middle East respiratory syndrome, caused by MERS-CoV in 2012–2013) showed evident sex-dependent differences in disease outcome (Karlberg et al., 2004). Notably, such a sex-dependent difference is presently observed in the new SARS pandemic, broken out in 2019 and caused by SARS-CoV-2 (COVID-19). In particular, susceptibility to SARS-CoV-2 infection is almost similar in both genders, but higher severity and mortality are observed in male patients (Wenham et al., 2020).

## Role of the “Cytokine Storm” in COVID-19

The previous severe acute respiratory syndromes caused by SARS-CoV and MERS-CoV were often associated with rapid viral replication, huge infiltration of inflammatory cells, and excessive production of proinflammatory cytokines (cytokine storm syndrome), leading to lung injury and respiratory distress syndrome (Channappanavar and Perlman, 2017). Notably, accumulating evidence demonstrates that cytokine storm syndrome is involved also in the most severe cases of COVID-19 (Mehta et al., 2020). These patients rapidly develop respiratory distress syndrome, lung edema and failure (often associated with hepatic, myocardial, and renal injury, hemostasis alteration). Elevated levels of proinflammatory cytokines are observed in these patients. In particular, compared with non-intensive care patients, intensive care patients have higher levels of IL-2, IL-7, and TNF. Many cytokines detected in these patients belong to the Th17 type response (as previously observed in MERS-CoV and SARS-CoV patients). The consequent IL-17-related pathway promotes broad pro-inflammatory effects by induction of specific cytokines, such as IL-1b, IL-6, TNF (responsible for systemic inflammatory symptoms), chemokines and matrix metalloproteinases (responsible for tissue damage and remodeling) (Wu and Yang, 2020). Moreover, pro-inflammatory cytokines, including IL-1b and IL-6, are directly induced by SARS-CoV-2 by interaction between viral components (probably nucleocapsid proteins) and toll like receptors of the host cells. Besides Th17 responses, patients diagnosed with COVID-19 showed marked rise of the Th1 subset (inflammatory cytokines IL-1β, IL-6, and IL-12) for more than 2 weeks after the infection onset (Russell et al., 2020).

In turn, IL-6 induced by SARS-CoV-2 in the lung seems to promote/amplify Th17 responses that may worsen the severe lung pathology in susceptible hosts (Hotez et al., 2020). In fact, IL-6 plays a crucial pathogenetic role in pulmonary injury induced by COVID-19. Accordingly, elevated levels of IL-6, produced by monocytes, lung interstitial fibroblasts, and alveolar macrophages, are observed in critical patients (Sun et al., 2020). Such a crucial role of IL-6 provided the rational basis for considering anti-IL-6 monoclonal antibodies (i.e. tocilizumab) as promising drugs for COVID-19 (Hotez et al., 2020).

## Estrogens in Cytokine Regulation

The complex pathways of cytokine regulation may pave the way to new pharmacological approaches aimed at limiting IL-6 expression and cytokine storm. As reported above, COVID-19 outcomes show clear gender-related differences; notably, gonadal hormones deeply influence the immune response. Indeed, estrogen receptors (ERs) regulate the expression of IL-6 gene through inhibition of transcription factors NF-IL6 and NF-κB, and through disruption of NF-κB transactivation (Luo and Zheng, 2016). As well, estradiol (and probably progesterone) inhibits Th17 cell differentiation (Chen et al., 2015). ERα activation in immune cells reduces Th1 and Th17 responses and skews cytokine production towards a Th2 type, with enhanced antibody response.

ER modulation has been proposed in a murine experimental model of pulmonary inflammation as a useful pharmacological strategy. In particular, ERα are expressed in resident and infiltrated inflammatory cells of the lungs and activation of these receptors by estradiol markedly reduces the histological and biochemical markers of inflammation. Notably, these effects were observed in both male and female animals (Vegeto et al., 2010). Protective effects of ER mediators were also observed in murine models of pulmonary inflammation induced by influenza virus infection (Vermillion et al., 2018). Consistently, estradiol (Zhang and Liu, 2020) and other estrogen hormones (such as the horse estrogen equilin) has been presently reviewed as an alternative option for the treatment of COVID‐19 (Suba, 2020).

## SERMs as Possible “Adjuvant Drugs” in COVID-19

Noteworthy, the protective effects evoked by endogenous estrogens are also promoted by drugs belonging to the class of SERMs (selective estrogen receptor modulators) (Polari et al., 2018). These drugs exhibit a complex profile of mixed agonist/antagonist modulators of the ER subtypes and their effects on immune system and immune-mediated inflammatory responses have been described (Behjati and Frank, 2009). Indeed, many preclinical and clinical studies demonstrated that SERMs evoke significant anti-inflammatory responses and inhibit the expression of many proinflammatory cytokines, in different conditions of systemic or local inflammation (Suuronen et al., 2005; Nalbandian et al., 2005; Cerciat et al., 2010; Azizian et al., 2018).

Concerning coronavirus infections, a single preclinical study investigated the role of sex hormones in shaping gender-related vulnerability to SARS-CoV. In this study, male and female mice were infected with murine-adapted SARS-CoV (Channappanavar et al., 2017). Male mice were more vulnerable to SARS-CoV infection compared to female mice. Such a higher susceptibility of male mice to SARS-CoV was associated with higher viral titers, enhanced vascular leakage, and alveolar edema. These changes were also associated with elevated levels of inflammatory cytokines in lungs of male mice. Ovariectomy or treatment of female mice with an ER antagonist increased mortality, indicating a protective effect for ER signaling in mice infected with SARS-CoV. In contrast, treatment of female mice with SERMs (i.e. tamoxifen) led to increased levels of protection.

Moreover, beyond the effects of SERMs on ERs, these drugs seem to present useful ancillary properties. Besides their potential effects on proinflammatory cytokine expression (mediated by ERs), some SERMs seem to play broader roles in inhibiting viral replication by ER-independent mechanisms. Indeed, *in vitro* studies on established cell lines reported that some drugs of the SERM class interfere with processes of viral entry into the host cell and inhibit different viral infections, including MERS-CoV, SARSCoV, and Ebola virus. These effects may be due to potential interaction with viral glycoproteins and with host proteins involved in the viral infection (Zhou et al., 2020).

**Figure 1:**
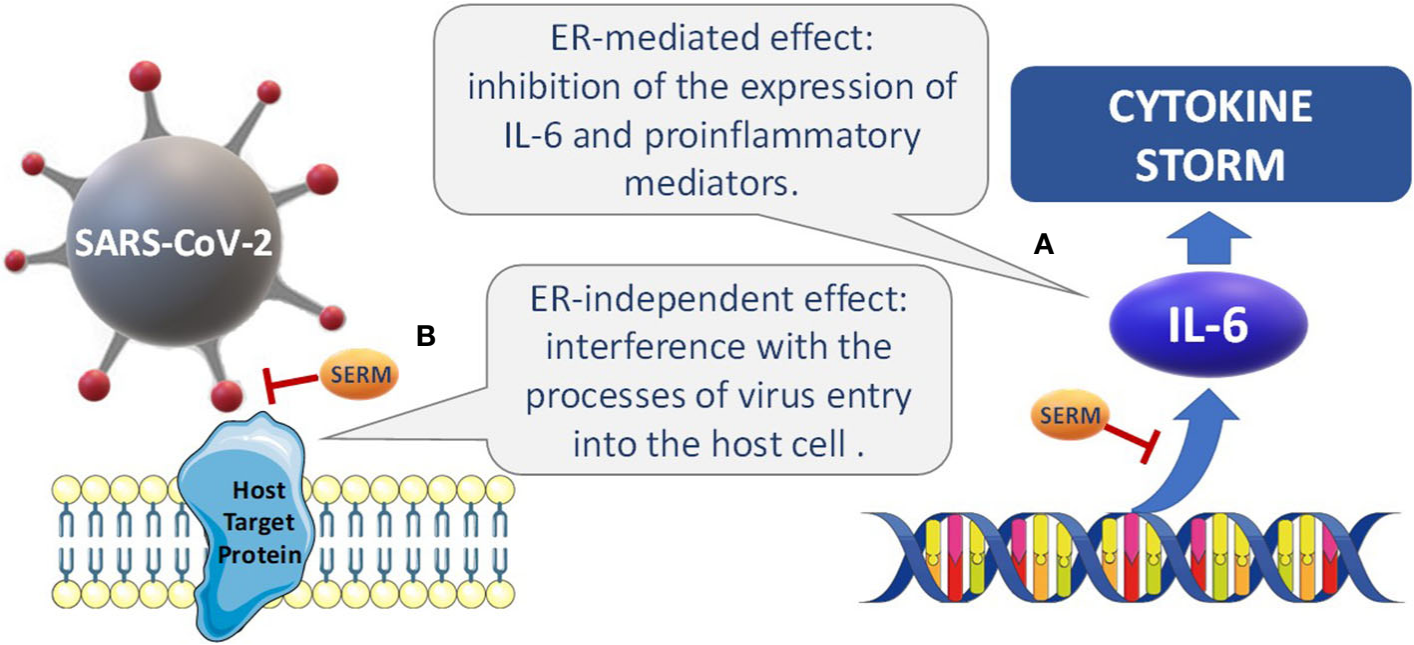
Hypothesized mechanisms accounting for the potential effects of SERMs. **(A)** ERs regulate the expression of proinflammatory cytokines, such as IL-6, by inhibition of the transcription factors NF-IL6 and NF-κB, and disruption of NFκB transactivation. **(B)** in experimental studies on established cell lines, some SERMs have been reported to interfere with the processes of viral entry into the host cell and to inhibit different viral infections, including MERS-CoV, SARSCoV, and Ebola. Potential interactions with viral glycoproteins and with host proteins involved in the viral infection have been hypothesized.

The hypothesized mechanisms of the potential effect of SERMs are summarized in **Figure 1**.

## Conclusion

Taken together, these data suggest that ER modulation may be a suitable pharmacological approach for preventing/attenuating the cytokine storm and inflammation associated with COVID-19 and in particular the use of SERMs and/or “tissue selective estrogen complex” (TSEC, i.e. association of SERM and natural estrogen) may represent a promising pharmacological option. Such a therapeutic approach would be particularly useful for treatment of both male and female patients in early phase of the disease (with mild/moderate symptoms), in order to prevent or mitigate the possible evolution towards more serious and dangerous forms of the disease, due to the onset of the cytokine storm.

## Author Contributions

All the authors equally contributed to the manuscript writing.

## Conflict of Interest

The authors declare that the research was conducted in the absence of any commercial or financial relationships that could be construed as a potential conflict of interest.
